# Acromio-Axillo-Suprasternal Notch Index Performance in Predicting Difficult Visualization of the Larynx: An Observational Prospective Study

**DOI:** 10.7759/cureus.66706

**Published:** 2024-08-12

**Authors:** Chhaya M Suryawanshi, Jayant Bhatia

**Affiliations:** 1 Anaesthesiology, Dr. D. Y. Patil Medical College, Hospital & Research Centre, Dr. D. Y. Patil Vidyapeeth (Deemed to be University), Pune, IND

**Keywords:** general anesthesia, difficult airway, laryngoscopy, difficult visualization of larynx, modified mallampati test, predictive value of tests, endotracheal intubation, acromio-axillo-suprasternal notch index

## Abstract

Bedside screening tests for predicting difficult intubation play a crucial role in clinical practice, although their utility remains limited. This prospective observational study aimed to assess the predictive value of the Acromio-Axillo-Suprasternal Notch Index (AASI) for difficult visualization of the larynx (DVL).

Following approval from the Institutional Ethics Sub-Committee (Research Protocol No.: IESC/FP/68/2023), this prospective, observational, single-center study involved a sample size of 100 consecutive adult patients, both male and female, aged 20 to 65 years. The participants were classified as American Society of Anesthesiologists (ASA) grade I or II and were scheduled for elective surgeries necessitating endotracheal intubation. Before the operation, the following factors were assessed: AASI, modified Mallampati test (MMT), sternomental distance (SMD), thyromental distance (TMD), and inter-incisor distance (IID). Larynx visualization was evaluated using the Cormack-Lehane (CL) grading system, where grades III and IV indicate difficult laryngeal visualization. Qualified anesthesiologists performed direct laryngoscopy while remaining unaware of the outcomes of the airway predictors being assessed.

The main aim of the investigation was to assess the efficacy of the AASI as a means of predicting DVL. The research study's secondary goals involved evaluating the accuracy of AASI in predicting challenging airways compared to other predictors such as MMT, SMD, TMD, and IID.

DVL was observed in 21% of patients, out of which 10 and 11 were males and females, respectively. The sensitivity, specificity, and area under the curve (AUC) of the AASI were observed and reported with a 95% confidence interval (CI), being 98.73% (93.2-99.9%), 71.43% (47.8-88.7%), and 0.851 (0.732-0.970), respectively. AUC analysis revealed that AASI outperformed MMT, SMD, TMD, and IID as a predictor of DVL.

AASI (≥0.5) serves as an excellent predictor for DVL during direct laryngoscopy. This finding suggests the clinical utility of AASI in identifying patients who may require special consideration during intubation procedures.

## Introduction

The American Society of Anesthesiologists (ASA) defines a difficult airway as "a clinical scenario where a physician with anesthesia training encounters anticipated or unanticipated challenges or inability to perform facemask ventilation of the upper airway, difficulty with tracheal intubation, or both" [1].

Managing a difficult airway is a critical aspect of healthcare, wherein experienced anesthesiologists may encounter complications when utilizing standard airway management techniques. The difficulty of airway management is highly variable and is determined by multiple aspects, including patient-specific attributes, medical history, surgical experience, airway evaluation, the clinical setting, and the patient's present physiological condition [2,3].

The prevalence of challenging laryngoscopy (Cormack grading III/IV) varies across different research. In investigations conducted on the Indian populace, Krishna et al. found that the incidence rate was 8.5% [4]. Conversely, among a community primarily composed of Kashmiri individuals, the risk of troublesome laryngoscopy was a mere 3.3% [5]. In a study conducted by Prakash et al., it was found that the frequency of complicated laryngoscopy was 9.7% without the use of external laryngeal pressure (ELP). However, when ELP was used, the incidence fell to 2.1% [6].

Assessment of the airway for indications of probable complications should be conducted promptly on any patient experiencing respiratory distress. Initially, it is important to conduct a prompt evaluation of genetic or acquired anatomic abnormalities. Special consideration should be given to individuals who have experienced facial, cranial, or cervical injuries, or who exhibit oral hemorrhaging, regurgitated stomach contents, or foaming at the mouth.

The modified Mallampati test (MMT) assesses the dimensions of the tongue, the sufficiency of mouth opening, and the state and existence of the teeth and uvula. The greater the visibility of the uvula located below the tongue, the more effortless the intubation process is expected to be. Externally, it is important to assess the thyromental distance (TMD). Intubation may become more challenging as the chin moves closer to the sternum while the head remains in a central position. Similarly, an extreme overbite can impede the proper positioning of a laryngoscope. Neck trauma can lead to an inability to securely move the head, and challenges with intubation may occur due to marginal placement. Obesity might also exacerbate airway difficulties. Excess tissue folds in the mouth and extra fat around the back of the throat can hinder the ability to see the vocal cords clearly [1].

Unforeseen difficulties in airway management can lead to severe complications, including decreased oxygen levels, greater chances of inhaling foreign substances, and displacement of the vocal cords. The patient may encounter extended periods of hoarseness, stridor, or an inability to effectively extubate due to blockage of the airway. Poor dental hygiene can create an environment where harmful microorganisms thrive, increasing the risk of pneumonia caused by inhaling bacteria or obstruction of the airways. Additional, infrequent problems may occur, including, tracheoesophageal fistula, as well as tracheal stenosis. In the end, hypoxic brain injury, cardiopulmonary arrest, and potentially death may occur.

Predicting the ease or difficulty of airway management requires a combination of appropriate training, clinical experience, risk assessment, and sound judgment. However, real-world assessments of airway difficulty can often be highly subjective even among experienced clinicians. It is therefore challenging to accurately predict which cases will present these unanticipated difficulties. Studies suggest that more than 90% of difficult airways are unanticipated [7].

Direct laryngoscopy has the potential to cause trauma and make the procedure more challenging. Therefore, it is crucial to establish a plan that maximizes the chances of successfully intubating on the first attempt, while also following rules in case of any failures or unexpected difficulties. There is no perfect method of evaluation, and unexpected challenges will occasionally arise. Efforts to identify an optimal predictor for difficult tracheal intubation have been an ongoing focus of research. Various predictors have been proposed over the years, yet none have achieved the sensitivity and specificity desired by practicing anesthesiologists.

Therefore, evaluating the airway is an essential component of patient assessment prior to administering anesthesia. Effective application of different tests, either individually or collectively, and careful assessment of how they may present as potential challenges, are crucial for devising strategies that can mitigate the anticipated or unanticipated problem and ensure a secure anesthesia procedure.

In this study, we explore the Acromio-Axillo-Suprasternal Notch Index (AASI) alongside common predictors such as the MMT, TMD, sternomental distance (SMD), and inter-incisor distance (IID) for predicting difficult visualization of the larynx (DVL).

This research endeavors to contribute to the understanding of accurate airway assessment, providing an evidence-based approach to identifying challenging airways, and ultimately improving the safety and effectiveness of airway management in clinical practice.

## Materials and methods

Prior to undertaking a prospective, observational, single-center study, the research got approval from the Institutional Ethics Sub-Committee (Research Protocol No.: IESC/FP/68/2023) and written informed consent was obtained. This study comprised a sample of 100 consecutive adult patients, aged between 20 and 65 years, who were classified as ASA grade I/II. These patients were scheduled to have elective procedures that required endotracheal intubation. The exclusion criteria encompassed patients who declined to participate, those with ASA grade III/IV, patients with structural abnormalities of the head, neck, and thorax, individuals using a cervical collar or with cervical spine abnormalities, patients with a history of head and neck surgery, obstetric patients, and individuals unable to open their mouths.

Each patient underwent a preoperative physical examination and an independent researcher assessed the following parameters: AASI, MMT, SMD, TMD, and IID.

**Figure 1:**
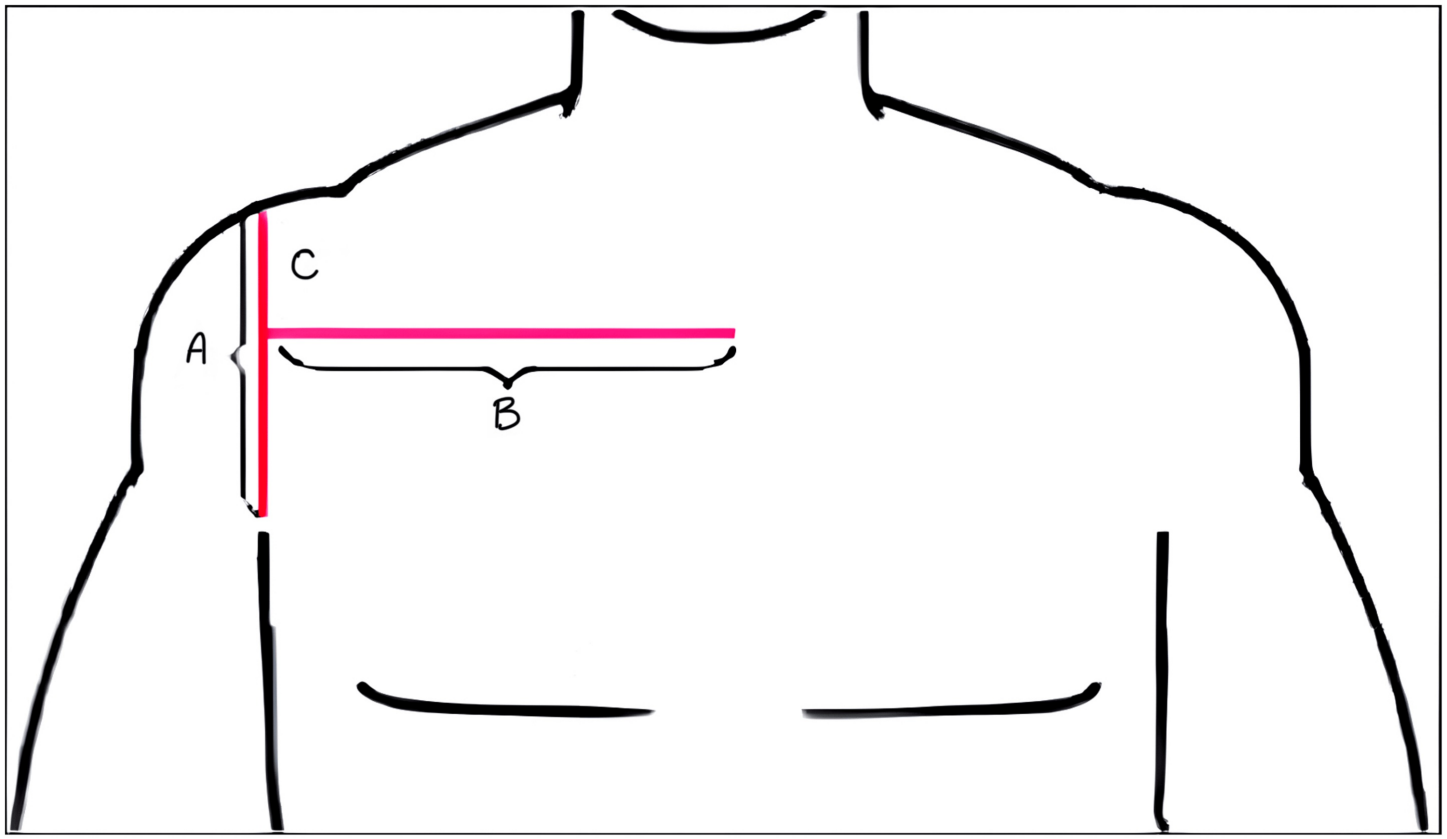
Measurement of the Acromio-Axillo-Suprasternal Notch Index (AASI). The measurement of the AASI involves several steps. First, a line is drawn from the top of the acromion process to the superior border of the axilla at the pectoralis muscle (A). Next, a perpendicular line is drawn from the suprasternal notch (B). The length of the segment of the line extending from the top of the acromion process to the point where it intersects with the perpendicular line is measured (C). Figure adapted under Creative Commons Attribution-NonCommercial-ShareAlike License (CC BY-NC-SA) from Senapathi TGA, Jobul IC, Widnyana IMG, Sucandra IMAK, Ryalino C, Raju A. Acromioaxillo suprasternal notch index as new screening test to predict difficult laryngoscopy in pediatric population: A case series. Bali J Anaesthesiol 2022;6:69-72. DOI: 10.4103/bjoa.bjoa_154_21.

For the AASI measurement (Figure 1), patients were positioned supine with their upper limbs resting alongside their bodies. Using a ruler, vertical and horizontal lines were drawn as line A and line B, respectively. The AASI was determined by the ratio of the length of line C to the length of line A. AASI greater than or equal to 0.5 was deemed indicative of challenging visualization of the larynx.

MMT consists of evaluating patients when they are sitting upright and keeping their heads in a neutral position. Patients were directed to optimize the extent of their mouth opening and completely extend their tongues without producing any sound. The observer assessed the anatomical structures of the pharynx and categorized them into classes I to IV according to the visible elements.

The TMD measurement was acquired by assessing the distance between the mentum (chin) and the thyroid notch with the patient's neck completely extended and their mouth closed. TMD values less than 6.5 cm were considered indicative of DVL.

SMD is the measurement of the direct distance between the manubrium sterni (the upper part of the sternum) and the mentum (chin). This measurement was taken when the head was fully extended and the mouth was closed. Values below 12.5 cm in SMD were seen as a sign of DVL.

IID was determined by measuring the gap between the upper and lower incisors when the mouth was fully open. IID values less than 4 cm were considered as a sign of DVL.

**Figure 2:**
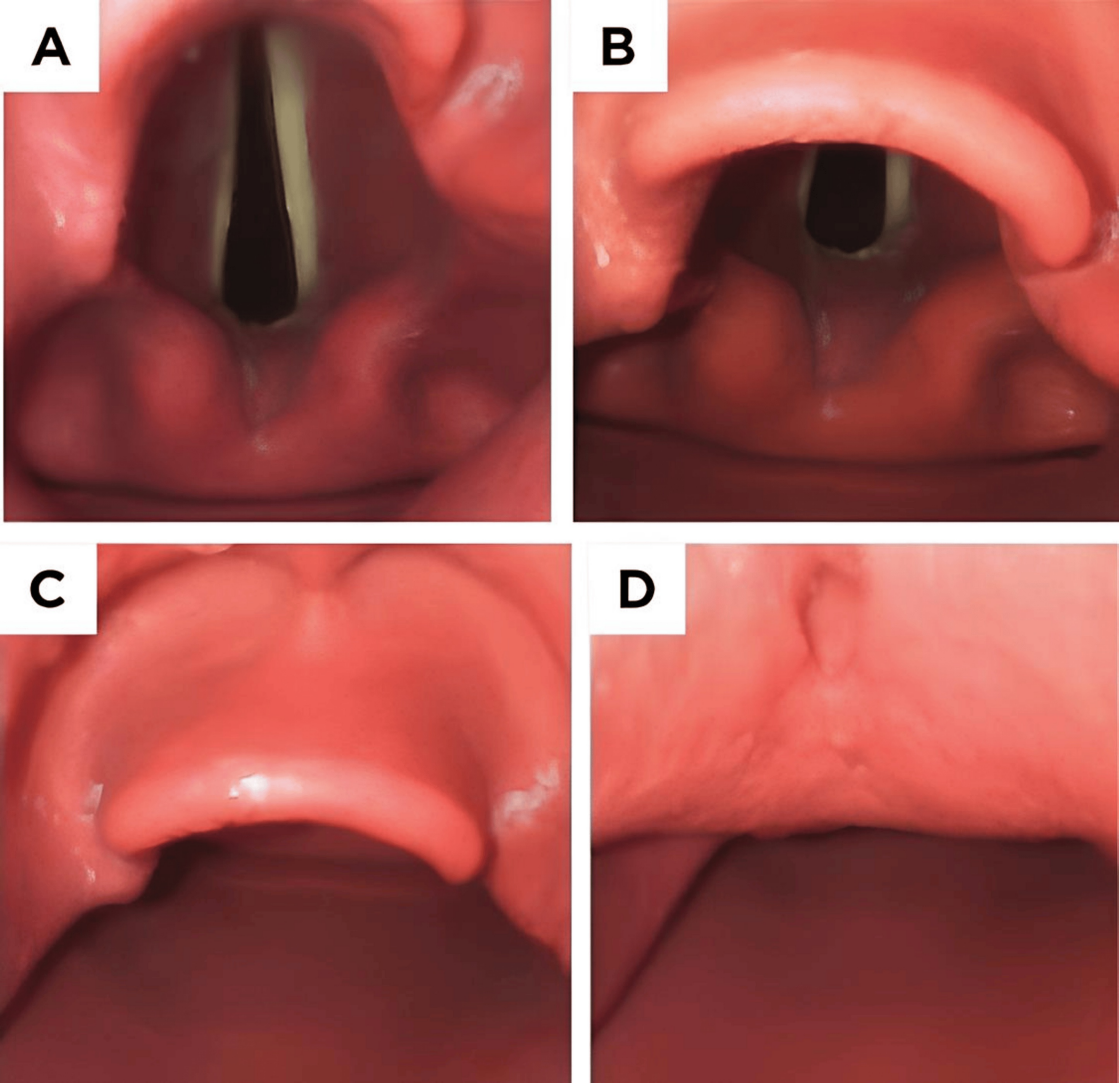
The Cormack-Lehane (CL) grading. The CL grading system for assessing laryngeal visibility during intubation includes four grades. Grade I represents full exposure of the glottis, including both the anterior and posterior commissures (A). Grade II indicates that the anterior commissure is not visible, although other parts of the glottis can be seen (B). Grade III is characterized by only the epiglottis being visible (C). Grade IV denotes that no glottic structures are visible, indicating a complete lack of visualization (D).

Every patient was premedicated with IV fentanyl at a dosage of 2 µg/kg. The anesthesia induction involved administering IV propofol 1% at a dose of 2 mg per kilogram of body weight. The dosage was adjusted until the patient no longer responded verbally. IV vecuronium bromide 0.1 mg/kg was administered to allow direct laryngoscopy and tracheal intubation. The head was then positioned in a sniffing morning air position and an expert anesthesiologist performed a laryngoscopy. Crucially, the person doing the laryngoscopy procedure did not have knowledge of the airway examinations conducted before the surgery. Patients' laryngeal views were graded with the Cormack-Lehane (CL) grading system (Figure 2).

Grades I and II are associated with easy visualization of the larynx (EVL), while grades III and IV are associated with DVL.

The statistical analyses were performed using Microsoft Excel (Microsoft Corporation, Redmond, WA) and inspected with the WINPEPI software (version 11.65). The chi-square test was employed to compare the percentages of qualitative variables. Continuous variables, including age, weight, AASI, MMT, TMD, SMD, and IID, were compared between groups using an unpaired t-test and were displayed as mean ± standard deviation. Gender, CL grading, EVL, and DVL were expressed as patient numbers and percentages, with Pearson's chi-square test for independence of attributes used to compare categorical groups. The sensitivity, specificity, positive predictive values, and negative predictive values of MMT, AASI, TMD, SMD, and IID, along with their 95% CI, were calculated, considering the laryngoscopic view as the gold standard. Additionally, the receiver operating characteristics (ROC) curves and the area under the curve (AUC) were developed for the airway predictors. Any p-value less than 0.05 was considered statistically significant at the 5% alpha level.

## Results

This study presents data for 100 patients. The demographic parameters are displayed in Table 1.

**Table 1:** Age, gender, and body weight of subjects categorized into the EVL and DVL groups. The mean and standard deviation of the quantitative variables (age and body weight) were calculated after verifying normal distribution. These values were then compared using a t-test. ^ Gender is presented as "n (%)"; * the reported values for EVL and DVL are expressed as mean ± standard deviation. EVL: easy visualization of the larynx; DVL: difficult visualization of the larynx.

Variables		EVL (n = 79)	DVL (n = 21)	p-value	Significance
Age (years)*		45 ± 13.3	52.2 ± 9.3	0.021	Significant
Gender^	Male	43 (54.5%)	10 (47.6%)	0.629	Not significant
	Female	36 (45.6%)	11 (52.4%)		
Body weight (kg)*		68.7 ± 14.6	83.5 ± 11.9	<0.001	Significant

Among all the cases, 53 were males. There was no statistically significant difference between the DVL and EVL groups in terms of demographic characteristics. Among the entire patient population, 21 individuals received a diagnosis of DVL, with females representing 52.4% of the cases. Among the individuals diagnosed with EVL, 54.5% were of the male gender.

Predictive values of airway predictors

The sensitivity and positive predictive value of all assessed airway predictive factors showed a wide range within the 95% CI. Of all the predictors, AASI showed the highest sensitivity, indicating the most negative predictive value. This suggests that there is a decreased probability that a participant with a negative test result would actually have a difficult airway (Table 2).

**Table 2:** The sensitivity, specificity, PPV, and NPV of the airway assessment tests. These tests were evaluated based on their sensitivity, specificity, positive predictive value (PPV), and negative predictive value (NPV), with their effectiveness quantified using confidence intervals (CI). Qualitative variables were analyzed by generating percentages and comparing them using the chi-square test. AASI: Acromio-Axillo-Suprasternal Notch Index; MMT: modified Mallampati test; TMD: thyromental distance; SMD: sternomental distance; IID: inter-incisor distance.

Airway assessment tests	Sensitivity (95% CI)	Specificity (95% CI)	PPV (95% CI)	NPV (95% CI)
AASI (≥0.5)	98.73% (93.2-99.9)	71.43% (47.8-88.7%)	77.6% (63.7-87.2)	98.3% (88.8-99.8)
MMT (≥III)	94.9% (87.5-98.6)	47.6% (25.7-70.2)	64.4% (54.6-73.2)	90.4% (76.6-96.4)
TMD (≤6.5 cm)	56.9% (45.3-68.1)	90.48% (69.6-98.8)	85.7% (61.2-95.8)	67.8% (63.9-82.02)
SMD (≤12.5 cm)	51.9% (40.4-63.3)	85.7% (63.7-96.9)	78.4% (55.5-91.4)	64.05% (57.2-70.4)
IID (≤4 cm)	49.4% (37.9-60.9)	85.7% (63.7-96.95)	77.6% (54.2-90.98)	62.86% (56.2-69.1)

ROC and AUC comparison

**Figure 3:**
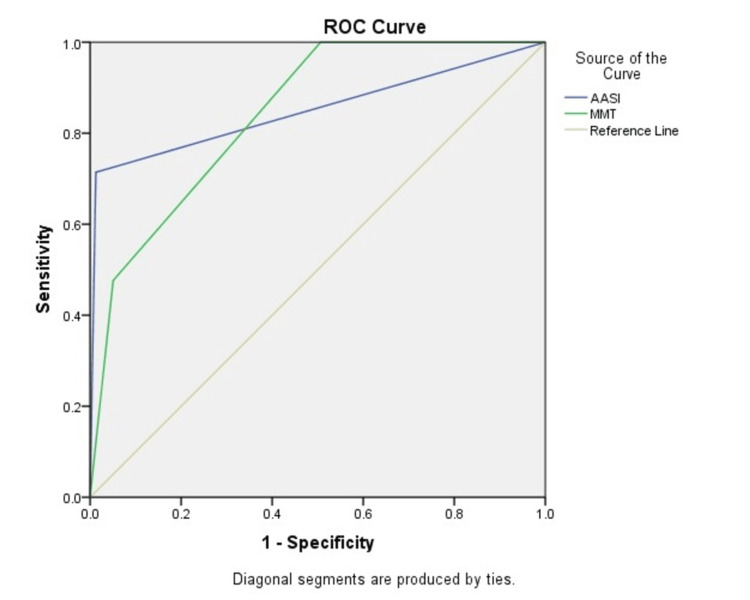
The ROC curve comparing AASI and MMT. ROC: receiver operating characteristics; AASI: Acromio-Axillo-Suprasternal Notch Index; MMT: modified Mallampati test.

Figure 3 presents a comparison of the ROC curves and AUC values for the AASI and the MMT.

**Figure 4:**
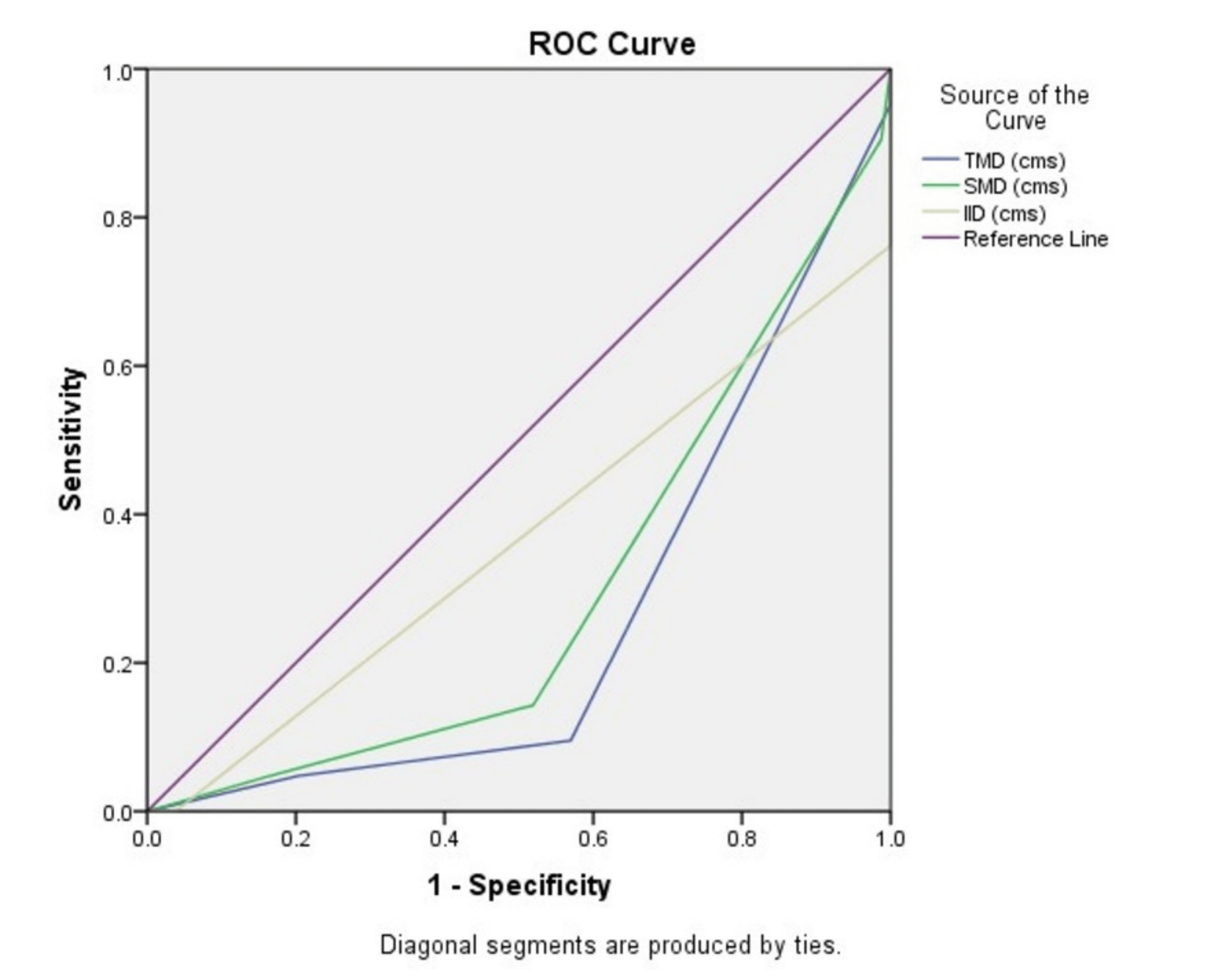
The ROC curve comparing TMD, SMD, and IID. ROC: receiver operating characteristics; TMD: thyromental distance; SMD: sternomental distance; IID: inter-incisor distance.

Figure 4 illustrates the comparison of ROC curves for TMD, SMD, and IID.

Table 3 summarizes the AUC values for the various airway assessment tests. The AASI attained the highest AUC at 0.851 among the predictors, while MMT had an AUC of 0.842.

**Table 3:** The AUC of ROC curves for AASI, MMT, TMD, SMD, and IID. AUC: area under the curve; ROC: receiver operating characteristics; AASI: Acromio-Axillo-Suprasternal Notch Index; MMT: modified Mallampati test; TMD: thyromental distance; SMD: sternomental distance; IID: inter-incisor distance.

Airway assessment tests	AUC	95% confidence interval
AASI	0.851	0.732-0.970
MMT	0.842	0.758-0.926
TMD	0.256	0.146-0.367
SMD	0.295	0.176-0.414
IID	0.366	0.221-0.512

## Discussion

Maintaining a patent airway is the paramount concern for anesthesiologists during the induction of general anesthesia. It directly impacts patient safety and successful surgery. To this end, numerous methods have been explored to predict difficult intubation during preoperative evaluation. These methods typically rely on straightforward physical examinations conducted at the patient's bedside, with a focus on anatomical landmarks. Some of the key tests used for this purpose include the MMT, TMD, SMD, and IID. While these tests have been widely employed, they have shown varying sensitivities and specificities.

The greater the sensitivity of a test, the lower the likelihood that a person with a negative test result would truly have a challenging airway, leading to an increased negative predictive value. As the test grows more specific, the likelihood of an individual with a positive test having a challenging airway reduces, hence increasing the positive predictive value. Therefore, the sensitivity of any analysis in this discipline should be enhanced while maintaining a suitable degree of specificity.

In light of these challenges, the study at hand explores a new avenue for predicting difficult intubation. Specifically, it investigates the region above the suprasternal notch, in the vicinity of the arm-chest junction, as a potential indicator of DVL. AASI, a novel parameter, emerges as a promising candidate for this purpose.

In the study conducted by Rajkhowa et al., the authors found that AASI was the most reliable predictor of DVL when compared to known measures such as MMT, SMD, TMD, and IID [8]. The study comprised a total of 440 individuals, with females accounting for 63% of the sample. Importantly, there were no substantial demographic disparities observed between the DVL and EVL groups. Out of the participants, 3.6% (16 persons) encountered DVL, with 56% of them being female. Conversely, 64% of the EVL group consisted of females. AASI had higher sensitivity (81.25 vs. 25) and positive predictive values (48.15 vs. 10.53) in comparison to MMT. Nevertheless, there were no significant variations observed in terms of specificity (96.7 vs. 91.98) and negative predictive value (99.27 vs. 97.01). The ROC and AUC values for AASI and MMT were 0.890 and 0.585, respectively. The ROC values for IID, SMD, and TMD were 0.571, 0.551, and 0.498, respectively. The ROC curves for MMT, SMD, TMD, and IID closely approximated the reference line. The AASI predictor had the maximum AUC value of 0.890. Our investigation also discovered that the sensitivity and negative predictive values of AASI were similar to those of MMT, SMD, TMD, and IID, as described in the aforementioned study. Although AASI had superior specificity and positive predictive values compared to MMT, TMD surpassed AASI and other airway tests in these characteristics. Significantly, AASI served as a distinctive indicator of the airway, as it assessed attributes that were not connected to the architecture of the head and neck.

Sateesh et al. conducted research focusing on the correlation between patient characteristics and laryngoscopy outcomes, involving 200 participants [9]. Their findings indicated no significant differences in weight, height, or BMI between patients with EVL and DVL. Notably, 6.3% of participants experienced difficult laryngoscopy. The study identified 0.49 cm as the optimal threshold for the AASI in predicting challenging intubation. Using this threshold, 76.9% of patients with difficult laryngoscopy were accurately identified, while 89.3% of those with no difficulties were correctly assessed. The AASI outperformed the MMT, showing higher positive predictive values (33.3 vs. 21.2) and a lower false-negative rate (4 vs. 7). The study found statistically significant differences in sensitivity (76.9 vs. 50), with the AASI being more accurate. However, there were no significant differences in specificity (89.3 vs. 86) and negative predictive values (98.2 vs. 95.8). The study reinforces the need for combining multiple existing tests due to the absence of a single definitive predictor for DVL.

Kamranmanesh et al.'s comprehensive study included 603 patients, of which 6.3% experienced difficult laryngoscopy [10]. Discriminant analysis identified 0.49 as the optimal AASI cutoff for predicting challenging intubation. The research highlighted that the AUC for AASI (0.89) was significantly higher than that of MMT (0.74). AASI's efficacy was evident, with 78.9% of patients with difficult laryngoscopy correctly identified and 89.4% of those without difficulties accurately predicted using this criterion. AASI excelled in terms of positive predictive values (33.3 vs. 21.6) and had a lower false negative rate (8 vs. 20) compared to MMT. Statistically significant differences were observed in sensitivity (78.9 vs. 52.4) and accuracy (88.7 vs. 83.4), favoring AASI. While there were no significant differences in specificity (89.4 vs. 85.7) and negative predictive values (98.4 vs. 96), the study suggested that AASI was a superior predictor for DVL, as also indicated in our study by its higher AUC (0.851 vs. 0.842). In conclusion, AASI was established as an effective predictor of DVL, surpassing MMT in terms of sensitivity, accuracy, and AUC.

However, it was emphasized in the study that no single test could have reliably predicted DVL, necessitating the use of multiple assessments in clinical practice. Given this, certain models were proposed that utilized a combination of many metrics, including IID, TMD, maximal range of head and neck mobility, MMT, and Naguib model (which incorporated thyrosternal distance, MMT, TMD, and neck circumference). Accordingly, it was recommended to conduct additional research in the future regarding contrasting AASI with other examinations.

Shanta et al.'s study involved 200 patients, 85% of whom were females [11]. The study found no significant demographic differences between the DVL and EVL groups. AASI exhibited higher sensitivity (62.5%) and predictive value (52.63%) compared to TMD with a sensitivity of 31.5% and a predictive value of 11.63%. In the comparison of AASI and TMD, AASI's AUC (0.79) significantly outperformed TMD's AUC (0.55). A threshold of greater than 0.5 was determined to be ideal by finding the point that achieved the best trade-off between false positives and genuine positives. The study found a prevalence of 8% for DVL in the patient population. Notably, significant differences were observed between the two tests in our study, with AASI having higher sensitivity (98.73 vs. 56.9) and negative predictive values (98.3 vs. 67.8) but lower specificity (71.43 vs. 90.48) and positive predictive values (77.6 vs. 85.7) compared to TMD. Both tests produced significantly different AUCs for ROC, with AASI at 0.851 and TMD at 0.256. This confirms AASI's superiority as a predictor of DVL. According to the study findings, AASI outperforms the traditional TMD test in terms of predictive values, including sensitivity, specificity, positive predictive value, and negative predictive value.

Chitralekha et al. conducted a prospective observational study with 100 patients categorized as ASA 1 and 2, aged between 18 and 50 years, who were scheduled for tracheal intubation in elective surgery [12]. The accuracy of AASI was evaluated in comparison to the modified Mallampati class (MMPC) and the ratio of neck circumference to thyromental distance (NC/TMD). Preoperative AASI, MMPC, and NC/TMD airway assessments were performed. After administering anesthesia, direct laryngoscopy was used to examine the larynx. The laryngeal view was recorded using the percentage of glottic opening (POGO) grading method. While comparing the positive predictive values, negative predictive values, sensitivity, and specificity of AASI, MMPC, and NC/TMD, AASI had a positive predictive value of 87%, MMPC had a positive predictive value of 80%, and NC/TMD had a positive predictive value of 21%. The negative predictive values were 85% for AASI, 85% for MMPC, and 68% for NC/TMD. The sensitivity values were 83% for AASI, 78% for MMPC, and 46% for NC/TMD. Lastly, the specificity values were 88% for AASI, 86% for MMPC, and 41% for NC/TMD. As a result, when compared to MMPC and NC/TMD, AASI has superior sensitivity, specificity, positive predictive values, and negative predictive values concluding that DVL can be predicted using AASI. Since there is no one test that can accurately predict DVL, AASI can be used to boost validity along with other common instruments like MMPC, TMD, and neck circumference.

In a study conducted by Girish et al., the predictive validity of a novel AASI was evaluated and compared with two established tests, the MMT and the TMD, for assessing difficult laryngoscopic views in accordance with the CL grading system [13]. This prospective, comparative, observational study included 250 adult patients, aged 18-65 years, of either sex, classified as ASA class 1, 2, and 3, who were scheduled for elective surgery under general anesthesia requiring tracheal intubation. Preoperative airway assessments were conducted using the AASI, TMD, and MMT. The study calculated sensitivity, specificity, positive predictive value, and negative predictive value for MMT, TMD, and AASI. The results indicated no significant difference between the MMT and the AASI in terms of sensitivity (58.1% vs. 67.7%), specificity (92.2% vs. 90%), positive predictive value (51.4% vs. 51.2%), and negative predictive value (94.0% vs. 95.2%) for predicting DVL.

In a study by Nasr-Esfahani et al., the AASI was evaluated as a method for predicting difficult tracheal intubation and laryngoscopy [14]. The study included 108 patients who required endotracheal intubation in the emergency department. According to the CL grading system, 54 patients had easy intubations (33.3% grade I and 66.6% grade II), while 52 patients experienced difficult intubations (57.7% grade III and 32.7% grade IV). Two patients were excluded due to the urgent need for treatment and their refusal to participate. At a cutoff point of 0.515, the sensitivity, specificity, positive predictive value, negative predictive value, and overall accuracy of AASI for predicting difficult intubation were 84.6%, 77.7%, 78.5%, 84%, and 81.13%, respectively, with an area under the ROC curve of 0.857. Thus, AASI is a highly accurate method with high sensitivity and specificity for predicting difficult endotracheal intubation.

The efficacy of the AASI in predicting difficult intubation was assessed by Shekhawat et al. [15]. The study also compared the AASI to the modified Mallampati test in the supine position (MMT-S). The study comprised a sample of 200 patients, ranging in age from 18 to 60 years, who had ASA physical status I and II. Patients who had structural problems in their head and neck, pregnant patients, patients who were missing all of their teeth, and those with a BMI greater than 35 kg/m² were not included in the study. Both the AASI and MMT-S were evaluated before the surgery. The AASI exhibited a sensitivity of 92% and a specificity of 97.71% in predicting difficult intubation, while the MMT-S showed a sensitivity of 76% and a specificity of 84.57%. The AUC for the AASI was 0.97. A cut-off value larger than 0.49 was found to be a reliable predictor of difficult intubation. The study found that AASI with a cut-off value over 0.49 had stronger sensitivity and positive predictive value, making it more successful than MMT-S in predicting a difficult airway in supine individuals.

The study by Jafarali Mulla et al. aimed to assess the predictive validity of the AASI for determining difficult laryngoscopic views [16]. This prospective, comparative, observational study included a calculated sample size of 250 patients. Among the 219 patients with EVL, 20 (9.1%) had an AASI greater than 0.5, while 199 (90.9%) had an AASI less than 0.5. Of the 31 patients with DVL, 21 (67.7%) had an AASI greater than 0.5, and 10 (32.3%) had an AASI less than 0.5. The sensitivity of AASI for predicting difficult laryngoscopic views was 67.7%, specificity was 90.9%, positive predictive value was 51.2%, and negative predictive value was 95.2%.

In their study, Bhaktavar et al. sought to compare the AASI, the ratio of height to thyromental distance (RHTMD), and the upper lip bite test (ULBT) as predictors of impaired laryngeal visualization before surgery [17]. A total of 240 adult patients with ASA physical status I and II, who needed general anesthesia for routine surgery, were included in the study. The airway of each patient was assessed using AASI, ULBT, and RHTMD. The main goal was to evaluate the effectiveness of AASI in predicting challenging airways. The secondary aim was to provide a comparison between AASI, RHTMD, and ULBT. Thirty-three patients were seen to have DVL, specifically classified as CL grades III and IV. The AASI showed superior performance compared to the ULBT in terms of sensitivity (93.94%), specificity (97.58%), positive predictive value (86.1%), and diagnostic accuracy (97.08%). Additionally, the AASI had a low false positive rate of 5%, while the ULBT had a sensitivity of 42.4%, specificity of 87.7%, positive predictive value of 35%, and diagnostic accuracy of 81.25%. The equivalent values for RHTMD were 75.76%, 47.34%, 18.66%, and 51.25%, respectively. These findings indicate that AASI outperforms both RHTMD and ULBT in predicting challenging laryngeal visualization. Ultimately, it was determined that a preoperative evaluation value of AASI over 0.49 is a strong and dependable indicator of challenging visibility of the larynx.

The study conducted by Sunkam et al. aimed to assess the accuracy of the AASI in predicting difficult laryngoscopy, comparing it with the ULBT and the MMT [18]. Additionally, the study examined the duration required to complete each test. The study encompassed a cohort of 150 patients who were slated to have elective surgery while under general anesthesia with endotracheal intubation. Preoperative airway assessments were conducted using AASI, ULBT, and MMT scores. AASI values equal to or greater than 0.49, ULBT class III, and MMT scores of III/IV were deemed indicative of DVL. Following the administration of anesthesia, the visibility of the larynx was assessed using the CL grading system. Calculations were performed to determine the sensitivity, specificity, predictive values, and accuracy of all three tests. A total of 18 individuals (12%) exhibited DVL (CL grades 3/4) according to the findings. The AASI demonstrated superior specificity (93.2%), positive predictive value (55%), and accuracy (89.3%) in comparison to both MMT and ULBT. The MMT test exhibited the maximum sensitivity rate of 77.8%, whilst ULBT had the lowest sensitivity rate of 50%. The duration required to execute AASI was greater (13.01 ± 1.03 seconds) in comparison to ULBT (7.49 ± 1.95 seconds) and MMT (3.97 ± 0.49 seconds). Ultimately, the MMT test proves to be the most responsive and expeditious method for forecasting DVL when compared to AASI and ULBT. Nevertheless, AASI demonstrates superior predictive ability for DVL as a result of its heightened specificity, PPV, and accuracy in comparison to conventional tests like MMT and ULBT.

Bamba et al. conducted a study to assess and compare the effectiveness of the AASI with the commonly used MMT in predicting difficult intubation [19]. The study was double-blind and prospective in nature. The study included 407 adult patients who had ASA physical status I and II and needed tracheal intubation for elective surgery. Airway assessments prior to surgery were performed using AASI and MMT. After administering general anesthesia, a skilled anesthesiologist assessed the direct observation of the larynx using the CL classification. A total of 28 patients (6.8%) exhibited DVL, namely, CL grades III and IV. The AASI, using a cutoff point of ≥ 0.5 cm, exhibited superior predictive values and lesser false values in comparison to MMT, hence enhancing its ability to detect DVL. The study determined that AASI (with a value of 0.5 cm or greater) is a valuable and dependable indicator of DVL in regular anesthetic procedures.

Collectively, these studies demonstrate that the AASI is an effective tool for predicting difficult laryngoscopy, with a distinct advantage over traditional measures. The AASI's unique approach, which goes beyond head and neck anatomy, and its remarkable sensitivity, accuracy, and AUC values make it a valuable addition to the anesthesiologist's toolkit. However, the complexity of DVL prediction necessitates a multifaceted approach, combining various tests. AASI represents a significant advancement in enhancing patient safety during intubation, highlighting its relevance in modern anesthesia practice.

Limitations

Various anesthesiologists performed the assessment of laryngeal views. Various factors, including technique, position during the procedure, and the height of the operating table, can impact the visibility of the larynx. Hence, there is a potential for inter-observer bias. To obtain a more precise estimate of sensitivity, it is necessary to conduct a study with a bigger sample size.

## Conclusions

The study concludes the utility of the AASI as a predictive tool for identifying DVL. It acknowledges that while no single test provides an entirely precise prediction of DVL, AASI can complement traditional tools such as the MMT, TMD, SMD, and IID, thereby enhancing the overall validity of preoperative assessments.

This approach recognizes the importance of combining multiple indicators to improve the accuracy and reliability of predicting difficult intubation. By integrating AASI into the existing battery of tests, anesthesiologists can achieve a more comprehensive and robust evaluation, ultimately leading to enhanced patient safety and better preparation for airway management during surgical procedures.
